# Alteration of fatty acid metabolism in the liver, adipose tissue, and testis of male mice conceived through assisted reproductive technologies: fatty acid metabolism in ART mice

**DOI:** 10.1186/1476-511X-12-5

**Published:** 2013-01-23

**Authors:** Li-Ya Wang, Fang Le, Ning Wang, Lei Li, Xiao-Zhen Liu, Ying-Ming Zheng, Hang-Ying Lou, Xiang-Rong Xu, Yun-Long Chen, Xiao-Ming Zhu, He-Feng Huang, Fan Jin

**Affiliations:** 1 Centre of Reproductive Medicine, Women’s Hospital, School of Medicine, Zhejiang University, Hangzhou, 310006, China; 2 Key Laboratory of Reproductive Genetics (Zhejiang), Ministry of Health, Hangzhou, 310006, China; 3 Department of Gynecologic Oncology, Henan Cancer Hospital, Zhengzhou, 450003, China; 4 College of Life Science, Zhejiang University, Hangzhou, 310058, China

**Keywords:** *In vitro* fertilization, Intracytoplasmic sperm injection, Fatty acid composition

## Abstract

**Background:**

Lipid metabolism plays important roles in the whole process of pregnancy. Previous studies have demonstrated abnormalities of lipid metabolism in the placentas of pregnancies obtained by assisted reproductive technology (ART). Therefore, we hypothesized that ART micromanipulation may affect lipid metabolism in offspring, and focused on the fatty acid metabolism in ART male offspring in this study.

**Methods:**

The fatty acid metabolism in the liver, adipose tissue and testis was detected. The comparison between naturally conceived (NC), controlled ovarian hyperstimulation (COH), *in vitro* fertilization (IVF) and intracytoplasmic sperm injection (ICSI) mice was made to analyze the effect of ART on offspring. The mice models in this study included two age groups: adult group and old group. The fatty acid composition and the expression of lipid metabolism-related genes were analyzed by GC-MS and qRT-PCR.

**Results:**

The fatty acid composition in the liver and adipose tissue were significantly altered in ART mice, but no significant difference was found in the testis. In adipose tissue, ART mice showed decreased monounsaturated fatty acids (MUFAs) and increased polyunsaturated fatty acids (PUFAs) in both adult and old mice, while the alteration of saturated fatty acids (SFAs) in the adult disappeared in the old. In liver, the changes were much complex in adult mice, while increased MUFAs and decreased PUFAs were found in ART old mice. The activities of fatty acid metabolism-related enzymes and the expression of lipogenic and lipolytic proteins changed in ART groups, with the adult mice and old mice showing inconsistent alterations. Further analysis indicated that SFAs was closely associated with the alterations of fatty acid metabolism-related enzyme activities and the expression of lipogenic and lipolytic proteins. Furthermore, we also found that the effect of separated ART treatments on fatty acid metabolism varied with different ages and tissues.

**Conclusions:**

ART treatments had effect on the fatty acid composition in adipose tissue and liver of male mice. The alteration of SFAs content was crucial for the regulation of fatty acid composition. These changes might have potential effects on the health of ART male offspring which need further investigation.

## Background

Assisted reproductive technologies (ART), such as *in vitro* fertilization (IVF) and intracytoplasmic sperm injection (ICSI), have been successfully applied to solve infertility problems in humans for several decades. However, their potential risks have been a subject of debate. Much evidence has demonstrated that IVF procedures could exert negative effects on developing fetus [1-4]. Pathological abnormalities in placentas associated with ART have also been investigated, including abnormal placental shape, abnormal umbilical cord insertion and increased placental weights [5,6]. Moreover, ART-induced impairment of placental steroid metabolism and restricted delivery of steroid hormones from mother to fetus have been reported [7].

Fatty acids are essential regulators in steroid hormone transportation and vital participants in cell signaling pathways [8,9]. The liver and adipose tissue play central roles in fatty acid metabolism. The liver acts as a major organ for fatty acid synthesis, while adipose tissue undertakes responsibility for triglyceride storage. The fatty acid composition of adipose tissue is an early determinant of childhood obesity [10], and abnormal lipid metabolism in abdominal adipose tissue may lead to many metabolic syndromes [11-13]. Kennedy A, Martinez K, Chuang CC, LaPoint K and McIntosh M [14] proposed that elevated saturated fatty acids (SFAs) could enhance adipose tissue expansion, which subsequently activated inflammatory signaling instead of insulin signaling to induce insulin resistance. Another research reported that abnormal fatty acid composition in atherosclerosis probably resulted from an increased fatty acid synthesis of monounsaturase activity [15]. Coronary heart disease and myocardial infarction in old men could be predicted by abnormal fatty acid composition [16,17]. In addition, the reproductive problems were also associated with fatty acid profile. Fatty acids have been shown to participate in oocyte maturation, fertilization, and embryo development [18-20]. Supplementation with polyunsaturated fatty acids (PUFAs) has been shown to delay gestation [21] and influence ovarian and uterine function [22-24]. Additionally, the fatty acid composition in the testis is also a key factor affecting spermatogenesis [25]. Either the decrease in docosahexaenoic acid (DHA, C22:6n-3) or the increase in SFAs have been detected in the sperm of oligozoospermic or asthenozoospermic men [26,27].

During fatty acid metabolism, the synthesis and catabolism of fatty acids are essential for regulating fatty acid composition. *De novo* lipid synthesis is regulated by fatty acid synthase (FAS) and its upstream ATP citrate lyase (ACLY) and acetyl-coenzyme A carboxylase alpha (ACACA). Carnitine palmitoyltransferase 1(CPT1), a mitochondrial enzyme, mediates the transport of long-chain fatty acids across the membrane during beta oxidation. The expression of CPT1 is associated with Type 2 diabetes and insulin resistance [28]. It was reported that the increase in fatty acid synthesis, together with the decrease of fatty acid oxidation suggested lipid accumulation [29,30]. The content of SFAs, monounsaturated fatty acids (MUFAs) and PUFAs can be regulated by a series of desaturases and elongases. Their activities were reported to be used for predicting metabolic diseases [31]. Stearoyl-CoA desaturase-1 (SCD1) is rate-limiting enzyme which regulates the balance between SFAs and MUFAs [32]. Long-chain fatty acids family member 6 (Elovl6) catalyzes the synthesis of saturated and monounsaturated fatty acids, and ELOVL6 deficiency may decrease the expressions of lipogeneic and lipid oxidation genes [33].

It was found that some male reproductive disorders occurred to ART offspring. For instance, boys conceived by IVF/ICSI had an increased risk of hypospadias or cryptorchidism and poor sperm quality in adulthood [34]. In addition, a decrease in serum testosterone and an increase in the LH/testosterone ratio occurred to three-month-old boys conceived by ICSI [35]. Adult man whose mothers received fertility treatment may have lower sperm count and concentration, smaller testis, and a lower free androgen index [36]. The problems in male ART offspring may arise from paternal inheritance or ART treatments. At present, whether ART methods could induce changes of fatty acid metabolism is still unclear. Our previous studies have found increased birth weight, but without abnormal semen quality and testicular morphology in ART mice models (Jin F *et al*., 2012 unpublished data). Therefore, in this research, we investigated the fatty acid metabolism in the adipose tissue, liver and testis of male ART mice, and analyzed the probable causes for the alterations. Our results provided the evidence of altered fatty acid metabolism in ART conceived mice.

## Results

### Fatty acid composition of the mouse diet

**Figure 1:**
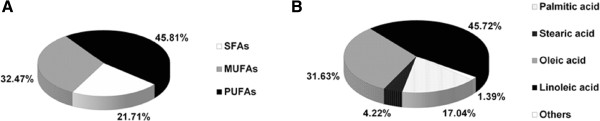
Fatty acid composition of the mouse diet.

In the mouse diet, SFAs, MUFAs and PUFAs accounted for 21.71%, 32.47%, and 45.81% of the total fatty acids respectively (Figure 1A). Oleic acid (C18:1n-9), linoleic acid (C18:2n-6), palmitic acid (C16:0) and stearic acid (C18:0) were the predominant fatty acids which accounted for 31.63%, 45.72%, 17.04% and 4.22% respectively (Figure 1B).

### Fatty acid composition in adipose tissue, liver and testis

In these three tissues, C16:0 and C18:0 were the two predominant SFAs. Palmitoleic acid (C16:1n-7) and C18:1n-9 were the main MUFAs. C18:2n-6 was the most abundant PUFA.

In adult testis, compared with NC group, only a little difference was found in ART groups, with a decreased eicosadienoic acid (C20:2n-9) in COH group and increased C14:0 and docosatetraenoic acid (C22:4n-7) in ICSI and IVF group respectively (Table 1). The total SFAs, MUFAs and PUFAs didn’t show significant difference between the four groups (data not shown).

**Table 1:** Fatty acid composition in adult testis

**Adult testis**	**Control**	**COH**	**IVF**	**ICSI**
c14:0	1.06 ± 0.30	1.07 ± 0.23	0.90 ± 0.21	1.75 ± 0.67^**^
c16:0	26.95 ± 3.59	28.15 ± 3.11	28.74 ± 3.04	26.02 ± 3.53
c18:0	6.73 ± 2.15	9.18 ± 3.67	6.00 ± 1.87	6.73 ± 2.19
c16:1n-7	3.83 ± 0.82	3.78 ± 0.54	3.56 ± 1.52	4.38 ± 1.57
c18:1n-1	0.05 ± 0.02	0.03 ± 0.01	0.06 ± 0.03	0.04 ± 0.01
c18:1n-9	25.15 ± 2.76	21.70 ± 3.57	24.19 ± 4.90	24.24 ± 2.77
c18:2n-6	25.31 ± 5.15	21.91 ± 6.53	19.79 ± 6.44	27.03 ± 7.32
c20:2n-9	0.58 ± 0.18	0.39 ± 0.12^*^	0.39 ± 0.09	0.47 ± 0.13
c20:3n-6	0.61 ± 0.25	0.60 ± 0.14	0.70 ± 0.33	0.49 ± 0.22
c20:4n-6	4.14 ± 1.14	4.98 ± 1.50	5.79 ± 2.84	3.89 ± 1.89
c22:4n-7	0.61 ± 0.23	1.09 ± 0.33	1.19 ± 0.40^*^	0.63 ± 0.37
c22:6n-3	4.99 ± 2.21	7.11 ± 2.46	8.68 ± 4.67	4.33 ± 3.59

The numbers reflect the percentages of total fatty acid methyl esters. The fatty acids whose content were more than 0.01% were shown in our data.^*^P < 0.05 *vs*. NC group; ^**^P < 0.01 *vs*. NC group. Data are presented as the means ± SD.

In adult adipose tissue, the ART groups contained higher levels (1.47-1.88 fold increase) of C14:0 and lower levels of C18:2n-6 (9.91-20.36% decrease) than NC group. Compared with COH group, increased C18:1n-9 and C22:4n-7 and decreased C18:0 and C18:2n-6 was found in IVF group. Higher content of C14:0 and C18:2n-6 and lower proportion of C22:4n-7 was detected in ICSI group compared with IVF group (Table 2).

**Table 2:** Fatty acid composition in adult adipose tissue

**Adult adipose tissue**	**NC**	**COH**	**IVF**	**ICSI**
C14:0	1.00 ± 0.12	1.47 ± 0.39	1.60 ± 0.18^*^	1.88 ± 0.77^*/b^
C16:0	20.05 ± 1.28	21.88 ± 1.01	22.17 ± 0.32	21.75 ± 1.90
C18:0	2.14 ± 0.26	2.64 ± 0.24	2.11 ± 0.22^a^	2.57 ± 0.57
C20:0	0.05 ± 0.01	0.05 ± 0.01	0.07 ± 0.02	0.07 ± 0.03
C14:1n-2	0.18 ± 0.04	0.14 ± 0.04	0.16 ± 0.01	0.17 ± 0.03
C16:1n-7	4.53 ± 0.85	5.45 ± 1.36	5.59 ± 0.56	5.01 ± 1.30
C16:1n-9	0.20 ± 0.03	0.23 ± 0.05	0.22 ± 0.002	0.21 ± 0.03
C18:1n-9	29.36 ± 3.54	29.97 ± 1.36	33.65 ± 0.45^*/a^	30.70 ± 2.33
C20:1n-9	0.50 ± 0.12	0.60 ± 0.06	0.79 ± 0.20	0.75 ± 0.22
C18:2n-6	40.28 ± 1.85	36.29 ± 0.99^**^	32.08 ± 0.87^**/aa^	35.44 ± 1.22^**/bb^
C18:3n-6	0.11 ± 0.03	0.10 ± 0.02	0.08 ± 0.02	0.07 ± 0.02
C20:2n-6	0.12 ± 0.01	0.11 ± 0.01	0.14 ± 0.04	0.15 ± 0.06
C20:3n-6	0.19 ± 0.02	0.16 ± 0.01	0.15 ± 0.04	0.15 ± 0.04
C20:4n-6	0.32 ± 0.05	0.25 ± 0.05	0.20 ± 0.07^*^	0.21 ± 0.06^*^
C20:5n-3	0.26 ± 0.03	0.23 ± 0.03	0.27 ± 0.06	0.24 ± 0.04
C22:4n-7	0.17 ± 0.01	0.12 ± 0.04^*^	0.26 ± 0.07^*/aa^	0.18 ± 0.06^b^
C22:6n-3	0.54 ± 0.02	0.33 ± 0.04^*^	0.47 ± 0.15	0.44 ± 0.15^*^

The numbers reflect the percentages of total fatty acid methyl esters. The fatty acids whose content were more than 0.01% were shown in our data.^*^P < 0.05 *vs*. NC group; ^**^P < 0.01 *vs*. NC group; ^a^*P* < 0.05 *vs*. COH group; ^aa^*P* < 0.01 *vs*. COH group; ^b^*P* < 0.05 *vs*. IVF group; ^bb^*P* < 0.01 *vs*. IVF group. Data are presented as the means ± SD.

In old adipose tissue, compared with NC group, ART mice contained lower levels of C14:0 (44.44-52.12% decrease), C14:1n-2 (47.37-63.16% decrease), C16:1n-9 (21.21-51.51% decreased), C18:2n-6 (11.10-14.14% decreased), linelenic acid (C18:3n-6) (66.67-83.33% decrease) and eicosapentaenoic acid (EPA, C20:5n-3) (55.56-66.67% decrease) and higher levels of C18:1n-9 (1.15-1.25 fold increase). More C18:1n-9 and less C16:1n-9 and C22:6n-3 was found in IVF group when compared to COH group. ICSI group presented higher content of C18:0, C20:1n-9, C22:4n-7 and C22:6n-3 than IVF group (Table 3).

**Table 3:** fatty acid composition in old adipose tissue

**Old adipose tissue**	**NC**	**COH**	**IVF**	**ICSI**
C14:0	1.17 ± 0.31	0.65 ± 0.14^**^	0.56 ± 0.07^**^	0.65 ± 0.15^**^
C16:0	17.11 ± 2.91	16.93 ± 0.85	16.71 ± 0.57	16.69 ± 2.59
C18:0	1.93 ± 0.90	1.89 ± 0.47	2.13 ± 0.41	3.26 ± 1.49^b^
C20:0	0.03 ± 0.02	0.04 ± 0.02	0.05 ± 0.04	0.11 ± 0.08^*^
C14:1n-2	0.19 ± 0.04	0.10 ± 0.02^**^	0.07 ± 0.03^**^	0.08 ± 0.02^**^
C16:1n-7	6.60 ± 1.91	6.32 ± 1.92	5.17 ± 1.35	3.65 ± 0.57^*^
C16:1n-9	0.33 ± 0.06	0.26 ± 0.02^*^	0.16 ± 0.04^**/aa^	0.19 ± 0.02^**^
C18:1n-9	33.17 ± 2.05	38.29 ± 0.76^**^	41.43 ± 1.85^**/a^	39.06 ± 2.12^**^
C20:1n-9	0.66 ± 0.23	0.64 ± 0.22	0.66 ± 0.24	1.23 ± 0.62^*/b^
C18:2n-6	37.70 ± 2.89	33.96 ± 1.55^*^	32.37 ± 1.48^**^	33.92 ± 2.07^*^
C18:3n-6	0.06 ± 0.02	0.02 ± 0.01^**^	0.01 ± 0.01^**^	0.02 ± 0.01^**^
C20:2n-6	0.05 ± 0.03	0.09 ± 0.02	0.08 ± 0.03	0.13 ± 0.06^**^
C20:3n-6	0.18 ± 0.03	0.13 ± 0.01	0.10 ± 0.02^*^	0.15 ± 0.04
C20:4n-6	0.18 ± 0.04	0.17 ± 0.03	0.16 ± 0.04	0.19 ± 0.12
C20:5n-3	0.18 ± 0.06	0.08 ± 0.01^**^	0.06 ± 0.03^**^	0.06 ± 0.04^**^
C22:4n-7	0.16 ± 0.05	0.14 ± 0.04	0.12 ± 0.03	0.22 ± 0.06^b^
C22:6n-3	0.30 ± 0.10	0.30 ± 0.01	0.17 ± 0.07^a^	0.39 ± 0.15^b^

The numbers reflect the percentages of total fatty acid methyl esters. The fatty acids whose content were more than 0.01% were shown in our data. ^*^P < 0.05 *vs*. NC group; ^**^P < 0.01 *vs*. NC group; ^a^*P* < 0.05 *vs*. COH group; ^aa^*P* < 0.01 *vs*. COH group; ^b^*P* < 0.05 *vs*. IVF group. Data are presented as the means ± SD.

**Figure 2:**
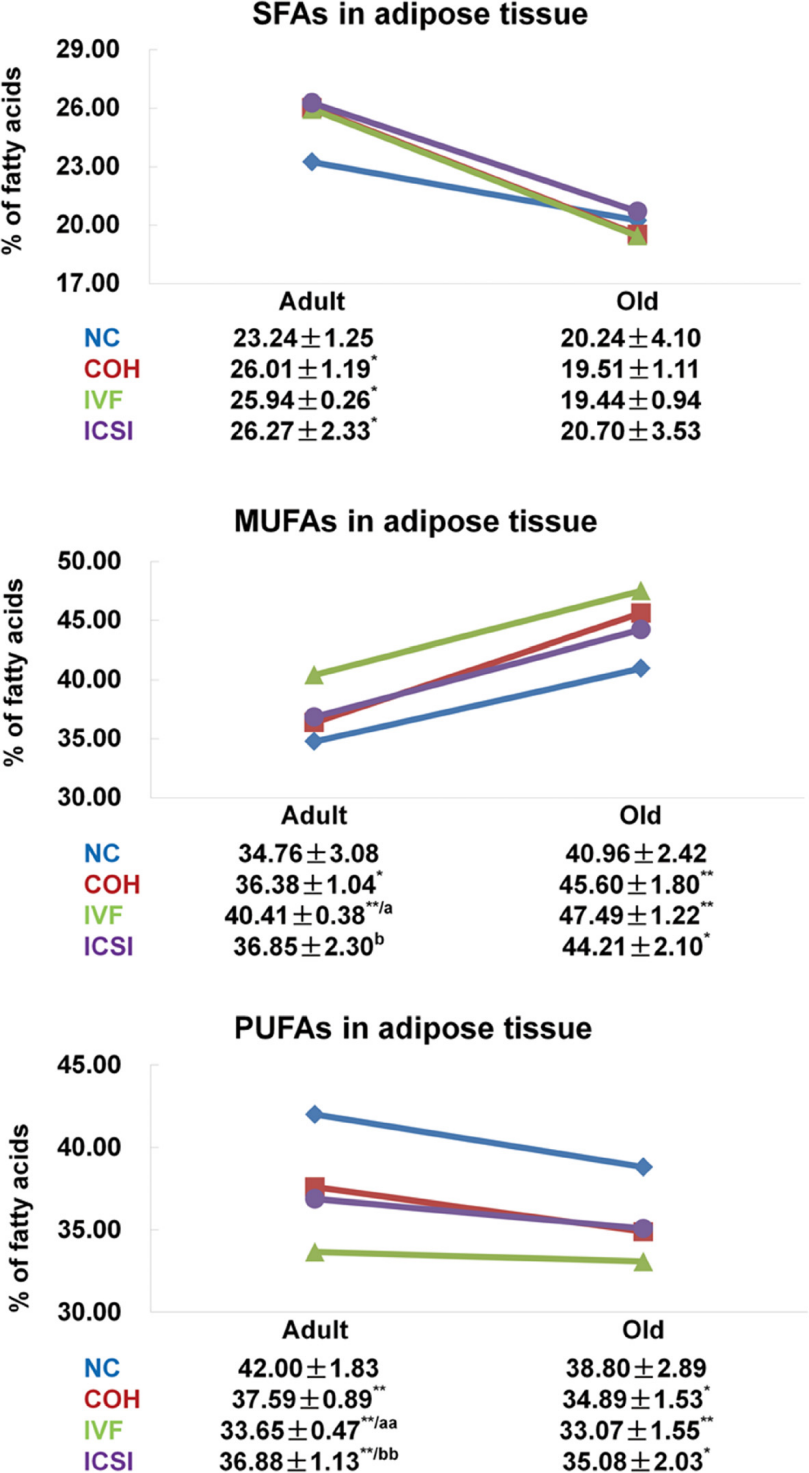
**The content of SFAs**, **MUFAs and PUFAs in adipose tissue of adult and old mice.** The numbers reflect the percentages of total fatty acid methyl esters. ^*^P < 0.05 *vs*. NC group; ^**^P < 0.01 *vs*. NC group; ^a^*P* < 0.05 *vs*. COH group; ^aa^*P* < 0.01 *vs*. COH group; ^b^*P* < 0.05 *vs*. IVF group; ^bb^*P* < 0.01 *vs*. IVF group. Data are presented as the means ± SD.

In adult adipose tissue, an up-regulated SFAs (1.12-1.13 fold increase) and MUFAs (1.05-1.16 fold increase) and a down-regulate PUFAs (10.5-19.88% decrease) existed in ART groups. IVF group had more MUFAs and less PUFAs than COH and ICSI group. In old adipose tissue, ART groups showed 1.08-1.16 fold increased MUFAs and 9.59-14.77% decreased PUFAs (Figure 2). The fatty acid composition in adult mice changed with age, and inconsistent variation of SFAs, MUFAs and PUFAs was shown in NC, COH, IVF and ICSI group.

In adult liver, C16:0, eicosatrienoic acid (C20:3n-6) and arachidonic acid (AA, C20:4n-6) were higher in COH group than NC group. Compared with COH group, an increased C16:0 and C22:4n-7 and a decreased C18:2n-6 and C20:4n-6 was found in IVF group. Moreover, ICSI group showed an up-regulated C16:1n-7 and down-regulated C20:4n-6, C22:4n-7 and C22:6n-3 compared with IVF group (Table 4).

**Table 4:** Fatty acid composition in adult liver

**Adult liver**	**NC**	**COH**	**IVF**	**ICSI**
C16:0	28.48 ± 1.59	25.19 ± 0.33^**^	29.32 ± 0.34^aa^	29.33 ± 0.94
C18:0	16.50 ± 0.87	17.37 ± 0.71	16.41 ± 0.99	15.14 ± 0.90^*^
C16:1n-7	0.56 ± 0.12	0.37 ± 0.07	0.67 ± 0.26	0.99 ± 0.27^**/b^
C18:1n-9	14.45 ± 2.30	12.83 ± 0.71	15.29 ± 1.37	17.69 ± 1.31^**^
C18:2n-6	21.70 ± 1.71	21.19 ± 1.29	17.39 ± 0.27^**/aa^	20.36 ± 1.84^b^
C20:3n-6	0.67 ± 0.15	0.91 ± 0.20^*^	0.89 ± 0.07	0.84 ± 0.18
C20:4n-6	8.91 ± 0.60	12.30 ± 0.89^**^	9.69 ± 0.31^aa^	7.94 ± 0.68^*/bb^
C22:4n-7	0.68 ± 0.11	0.73 ± 0.11	0.97 ± 0.15^**/a^	0.73 ± 0.16^b^
C22:6n-3	8.05 ± 3.68	9.11 ± 0.70	9.38 ± 1.30	6.98 ± 0.76^b^

The numbers reflect the percentages of total fatty acid methyl esters. The fatty acids whose content were more than 0.01% were shown in our data. ^*^P < 0.05 *vs*. NC group; ^**^P < 0.01 *vs*. NC group; ^a^*P* < 0.05 *vs*. COH group; ^aa^*P* < 0.01 *vs*. COH group; ^b^*P* < 0.05 *vs*. IVF group; ^bb^*P* < 0.01 *vs*. IVF group. Data are presented as the means ± SD.

In old liver, the difference between NC and ART groups mainly concentrated on COH and IVF group which had lower content of C16:0 and higher content of C18:1n-9, and the content of all PUFAs (except C18:2n-6) was lower than NC group. ICSI group showed higher proportion of C18:0 and lower proportion of C20:4n-6, C22:4n-7 and C22:6n-3 than NC group. Compared with COH group, only C22:4n-7 and C22:6n-3 had decreased content in IVF group. The fatty acid composition in ICSI group was totally different from that in IVF group except C16:1n-7and C18:2n-6 (Table 5).

**Table 5:** Fatty acid composition in old liver

**Old liver**	**NC**	**COH**	**IVF**	**ICSI**
C16:0	41.33 ± 1.30	35.01 ± 1.18^**^	32.53 ± 4.65^**^	39.15 ± 4.67^b^
C18:0	14.03 ± 1.87	13.11 ± 2.31	11.42 ± 3.99	19.31 ± 3.38^*/bb^
C16:1n-7	1.84 ± 0.59	1.50 ± 0.34	1.99 ± 0.99	1.32 ± 0.52
C18:1n-9	16.07 ± 2.52	30.21 ± 2.25^**^	33.87 ± 3.26^**^	19.86 ± 0.68^bb^
C18:2n-6	12.21 ± 1.20	14.26 ± 2.23	15.47 ± 5.32	10.52 ± 1.70
C20:3n-6	1.37 ± 0.15	0.71 ± 0.09^**^	0.63 ± 0.25^**^	1.32 ± 0.07^bb^
C20:4n-6	9.28 ± 0.76	3.86 ± 0.56^**^	3.36 ± 1.31^**^	6.76 ± 0.36^**/bb^
C22:4n-7	0.98 ± 0.10	0.29 ± 0.04^**^	0.16 ± 0.04^**/aa^	0.39 ± 0.03^**/bb^
C22:6n-3	2.89 ± 0.34	1.04 ± 0.14^**^	0.56 ± 0.13^**/a^	1.37 ± 0.11^**/bb^

The numbers reflect the percentages of total fatty acid methyl esters. The fatty acids whose content were more than 0.01% were shown in our data. ^*^P < 0.05 *vs*. NC group; ^**^P < 0.01 *vs*. NC group; ^a^*P* < 0.05 *vs*. COH group; ^aa^*P* < 0.01 *vs*. COH group; ^b^*P* < 0.05 *vs*. IVF group; ^bb^*P* < 0.01 *vs*. IVF group. Data are presented as the means ± SD.

**Figure 3:**
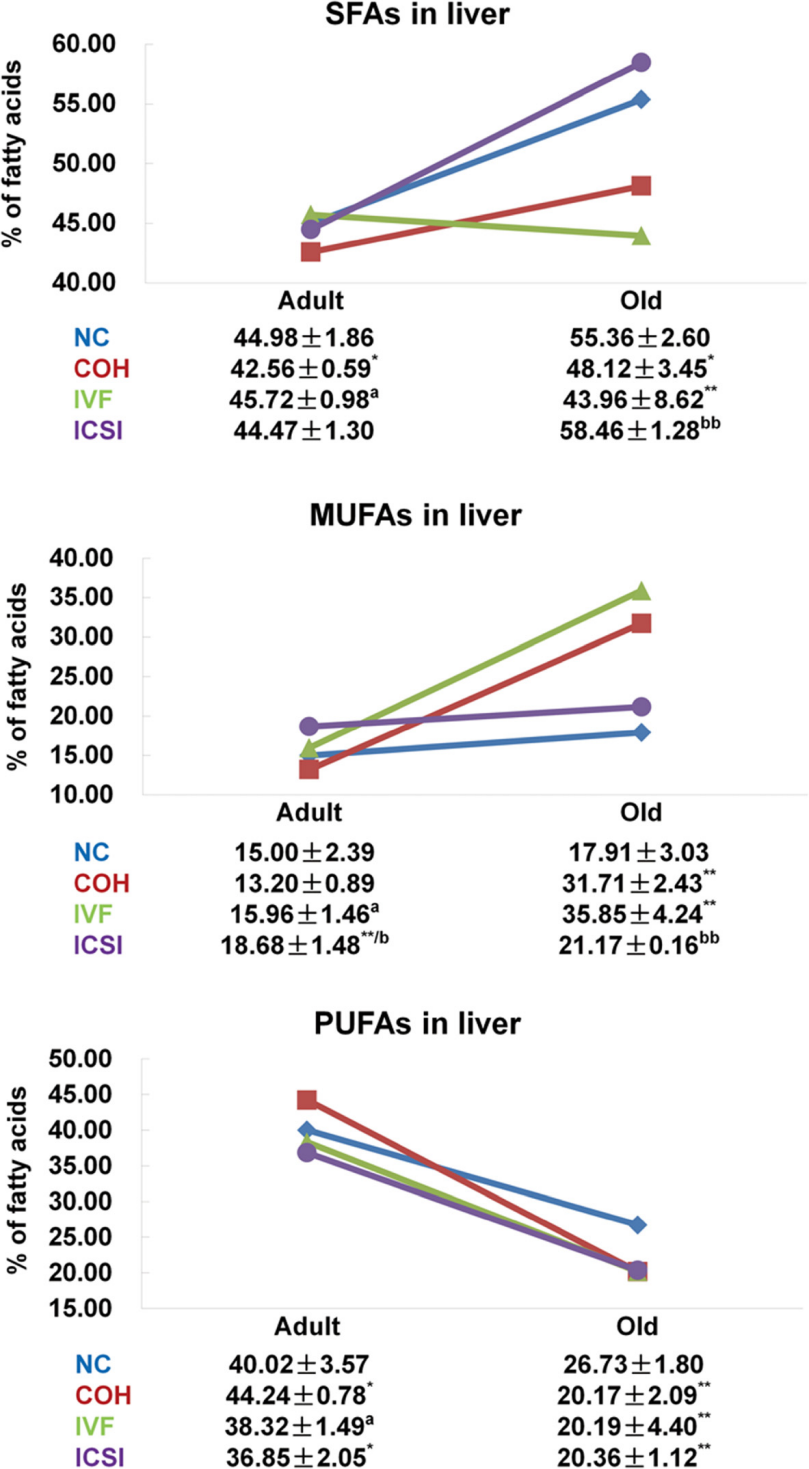
**The content of SFAs, MUFAs and PUFAs in the liver of adult and old mice.** The numbers reflect the percentages of total fatty acid methyl esters. ^*^P < 0.05 *vs*. NC group; ^**^P < 0.01 *vs*. NC group; ^a^*P* < 0.05 *vs*. COH group; ^b^*P* < 0.05 *vs*. IVF group; ^bb^*P* < 0.01 *vs*. IVF group. Data are presented as the means ± SD.

For the total content of SFAs, MUFAs and PUFAs in adult liver, COH group had a lower content of SFAs and a higher content of PUFAs than NC group, while ICSI group contained increased MUFAs and decreased PUFAs. Compared with COH group, up-regulated SFAs and MUFAs and down-regulated PUFAs was found in IVF group. The difference between IVF and ICSI group was only represented in the content of MUFAs. In old liver, COH and IVF group contained more MUFAs and less SFAs and PUFAs than NC group, but no difference existed between COH and IVF group. ICSI group showed higher content of SFAs than IVF group and lower level of MUFAs than NC and IVF group (Figure 3). The fatty acid composition in adult mice changed with age, and inconsistent variation of SFAs, MUFAs and PUFAs was shown in NC, COH, IVF and ICSI group.

### The activities of fatty acid metabolism-related enzymes

**Figure 4:**
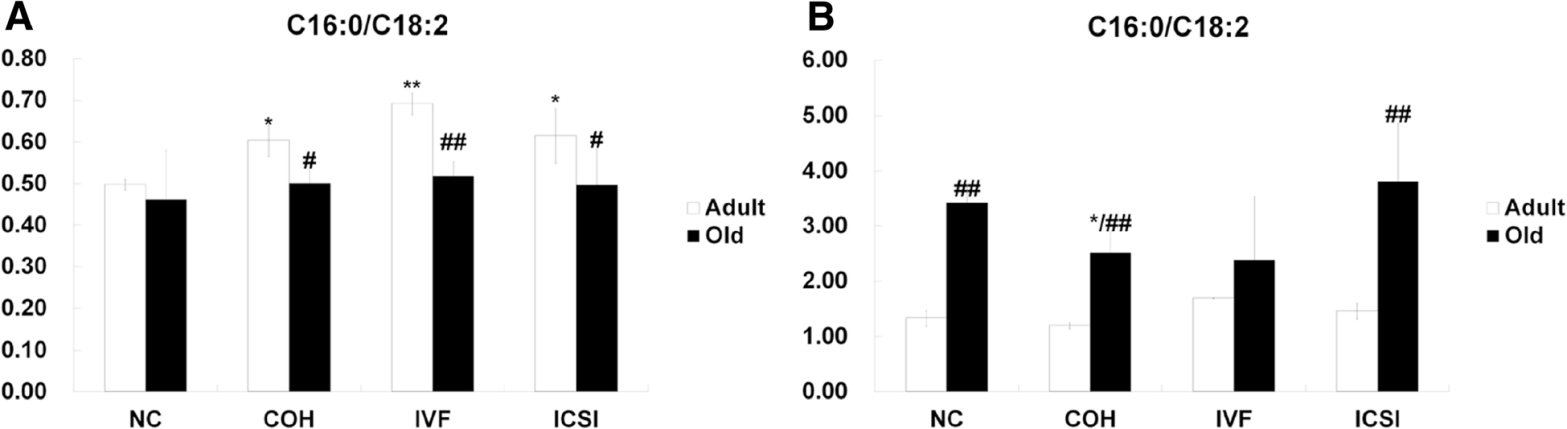
**The *de novo *synthesis index in adipose tissue and liver.** (**A**) C16:0/C18:2 in adipose tissue of adult and old mice. (**B**) C16:0/C18:2 in liver of adult and old mice. ^*^P < 0.05 *vs*. corresponding NC group; ^**^P < 0.01 *vs*. corresponding NC group; ^#^*P* < 0.05 *vs*. corresponding Adult group; ^##^*P* < 0.01 *vs*. corresponding Adult group.

In adipose tissue, C16:0/C18:2 in three adult ART groups were higher than that in NC group. C16:0/C18:2 in ART groups decreased with age (Figure 4A). In liver, C16:0/C18:2 showed no difference between the four adult groups. C16:0/C18:2 in old COH group was lower than that in NC group, and the ratio in NC, COH and ICSI groups increased with age (Figure 4B).

**Figure 5:**
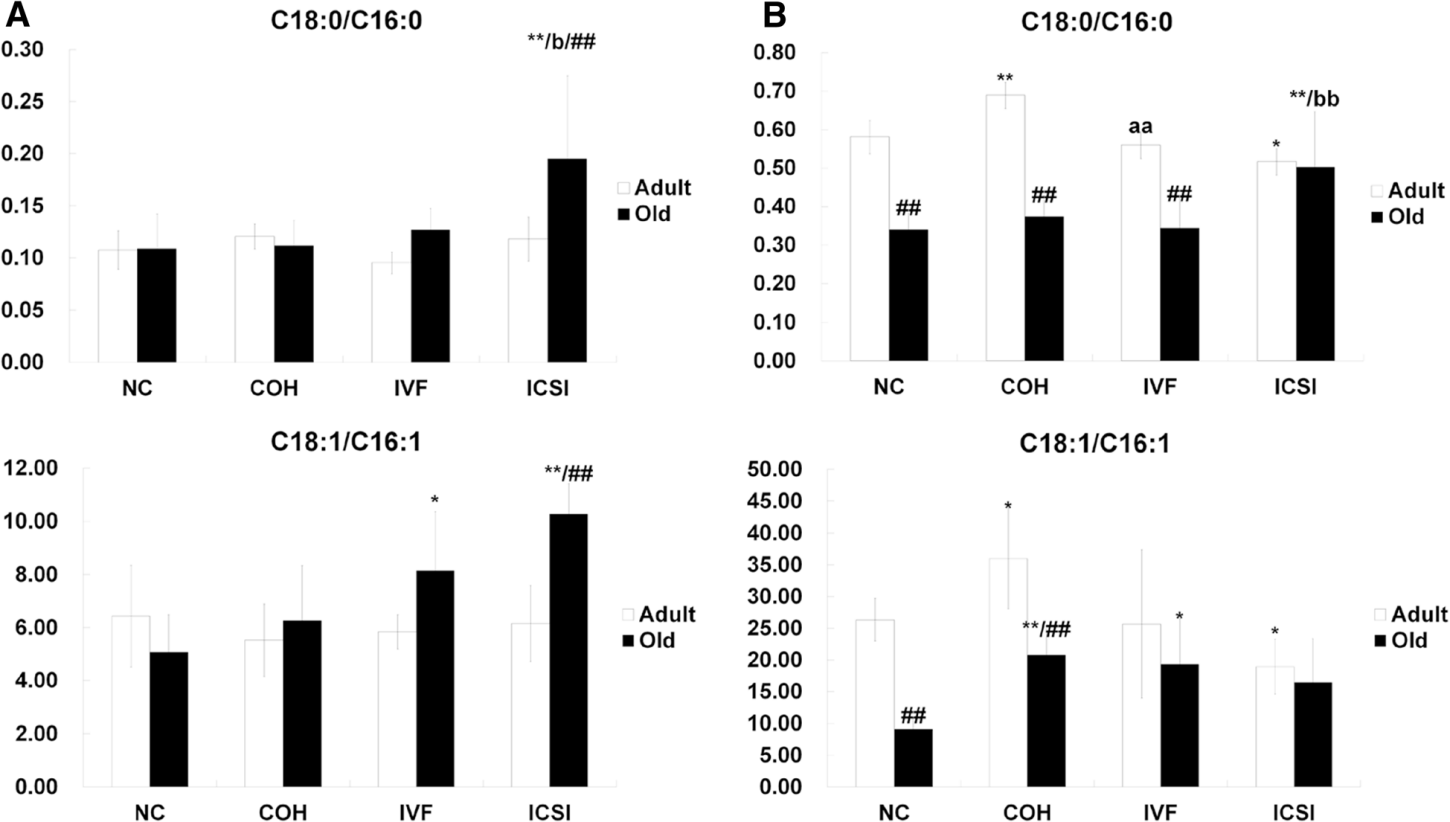
**The estimated ELOVL6 activities in adipose tissue and liver.** (**A**) C18:0/C16:0 and C18:1/C16:1 in adipose tissue of adult and old mice. (**B**) C18:0/C16:0 and C18:1/C16:1 in liver of adult and old mice. ^*^P < 0.05 *vs*. corresponding NC group; ^**^P < 0.01 *vs*. corresponding NC group; ^##^*P* < 0.01 *vs*. corresponding Adult group; ^aa^*P* < 0.01 *vs*. corresponding COH group; ^b^*P* < 0.05 *vs*. corresponding IVF group; ^bb^*P* < 0.01 *vs*. corresponding IVF group.

In adipose tissue, C18:0/C16:0 in old ICSI group was significant higher than that in NC and IVF old group. C18:1/C16:1 was up-regulated in old IVF and ICSI group compared to NC group. Both C18:0/C16:0 and C18:1/C16:1 in ICSI group increased with age (Figure 5A). In liver, C18:0/C16:0 in adult COH group was higher than that in NC and IVF group. C18:0/C16:0 in adult ICSI group was lower than that in NC group. ICSI old group showed increased C18:0/C16:0 compared with NC and IVF group. C18:0/C16:0 in NC, COH and IVF groups were down-regulated with age. Compared with adult NC group, C18:1/C16:1 was higher in COH group and lower in ICSI group. COH and IVF old group presented enhanced C18:1/C16:1 in comparison with NC group. C18:1/C16:1 in NC and COH group decreased with age (Figure 5B).

**Figure 6:**
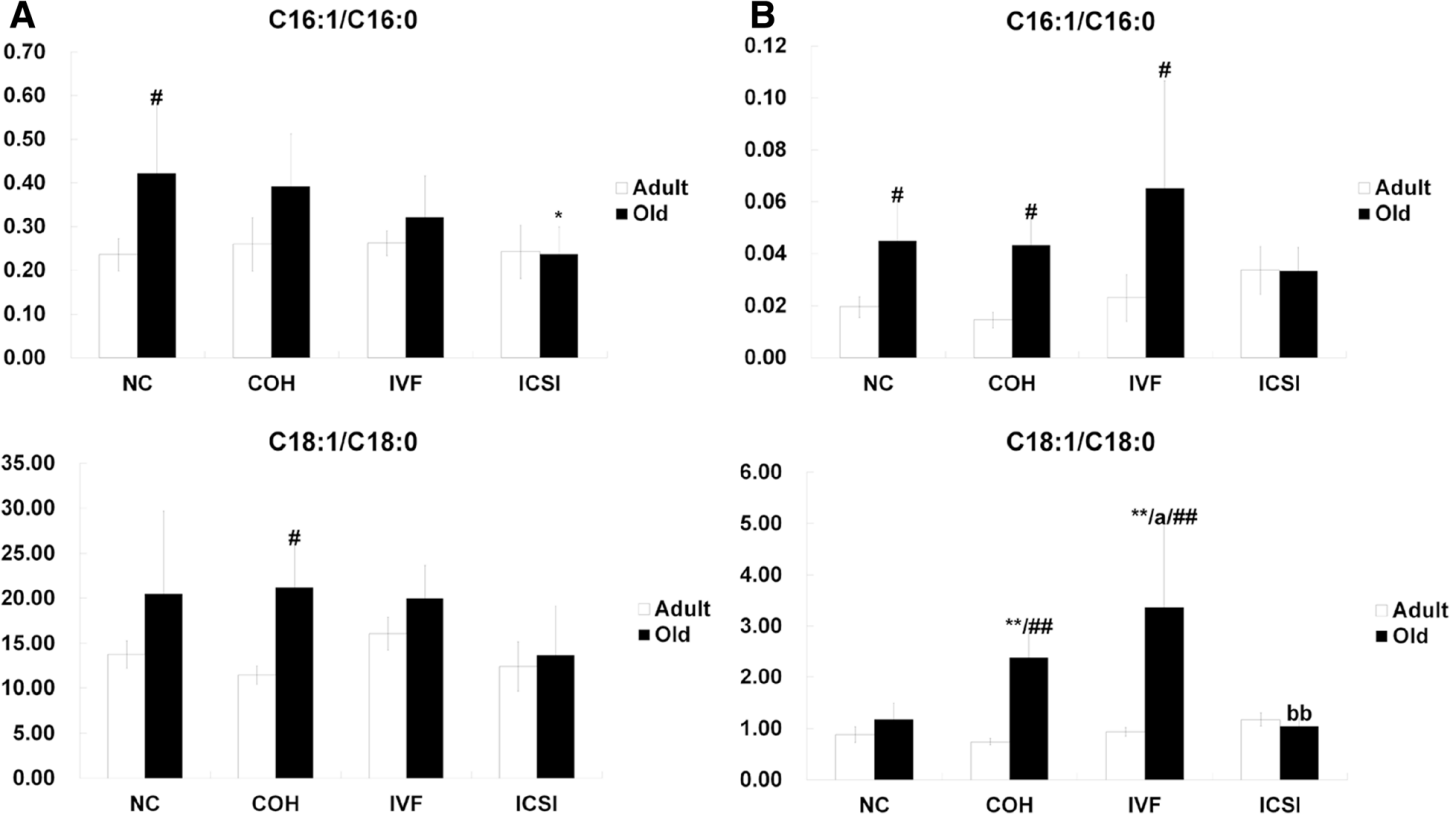
**The estimated SCD1 activities in adipose tissue and liver.** (**A**) C16:1/C16:0 and C18:1/ C18:0 in adipose tissue of adult and old mice. (**B**) C16:1/C16:0 and C18:1/ C18:0 in liver of adult and old mice. ^*^P < 0.05 *vs*. corresponding NC group; ^**^P < 0.01 *vs*. corresponding NC group; ^#^*P* < 0.01 *vs*. corresponding Adult group; ^##^*P* < 0.01 *vs*. corresponding Adult group; ^a^*P* < 0.05 *vs*. corresponding COH group; ^bb^*P* < 0.01 *vs*. corresponding IVF group.

In adipose tissue, C16:1/C16:0 in old ICSI group was lower than that in NC group. C16:1/C16:0 in NC group was down-regulated with age, while C18:1/C18:0 in COH was up-regulated with age (Figure 6A). In liver, C16:1/C16:0 in NC, COH and IVF groups increased with age, but no difference was shown between the four groups in the same age group. C18:1/C18:0 in old COH and IVF group was up-regulated compared with NC group. Old IVF group presented higher C18:1/C18:0 than COH and ICSI group. C18:1/C18:0 in COH and IVF group increased with age (Figure 6B).

### Correlation analysis of lipid metabolism in adult and old mice

To investigate the influence of varied fatty acid metabolism-related enzyme activities on the fatty acid composition, we analyzed the correlation between fatty acids proportion and the activities of lipid metabolism-related enzymes. Their correlation weren’t always consistent between two age groups in both adipose tissue and liver. For instance, in liver, the correlation between C16:0/C18:2 and SFAs, C16:0/C18:2 and PUFAs, and MUFAs and C18:1/C18:0 were consistent in adult and old mice, while the correlation between the estimated SCD1 activities and SFAs were inconsistent. Furthermore, the opposite state between two ages was also existed in liver, such as the correlation between MUFAs and C16:0/C18:2n-6, MUFAs and C18:1/C16:1, PUFAs and C18:0/C16:0, and between PUFAs and C16:1/C16:0 (Additional file 1: Table S1).

To analyze the crucial factor that resulted in the inconsistent correlation, especially the opposite correlation mentioned above, the relationship between all fatty acid and ELOVL6, SCD1 activities and *de novo* synthesis index were detected in both adult and old mice. In liver, the correlation between C18:1n-9 and C18:1/C16:1, C20:4n-6 and C18:1/C16:1, and between C16:1n-7 and C16:0/C18:2n-6 in adult mice was opposite to that in old mice. Coincidentally, the contrary relationship also existed between these three fatty acids (C16:1n-7, C18:1n-9 and C20:4n-6) and C16:0 (Table 6). In adipose tissue, although we didn’t found the opposite state between two age groups when analyzing the correlation between the activities of lipid metabolism-related enzymes (Δ5 and Δ6 desaturases were also analyzed, data were not shown) and SFAs, MUFAs or PUFAs (Additional file 1: Table S1), the contrary relationship between these enzymes activities and individual PUFAs were found. These individual PUFAs (C20:2n-6, C20:4n-6 and C22:4n-7) were positively related to C18:0 in adult adipose tissue, but negatively associated with C18:0 in old adipose tissue (Table 7).

**Table 6:** **The relationship between estimated ELOVL6 and SCD1 activities**, ***de novo *synthesis index, C16:0, C16:1n-7, C18:1n-9 and C20:4n0-6 in liver**

**Liver**	**Estimated ELOVL6 activity**				**Estimated SCD1 activity**				**C16:0/C18:2n-6**		**C16:0**	
	**C18:0/C16:0**		**C18:1/C16:1**		**C16:1/C16:0**		**C18:1/C18:0**					
	**Adult**	**Old**	**Adult**	**Old**	**Adult**	**Old**	**Adult**	**Old**	**Adult**	**Old**	**Adult**	**Old**
C16:1n-7	−0.797^**^	−0.718^**^	−0.865^**^	−0.658^**^	0.970^**^	0.946^**^	0.887^**^	0.517^**^	0.466^*^	−0.602^**^	0.514^*^	−0.485^**^
C18:1n-9	−0.735^**^	−0.096	−0.508^*^	0.589^**^	0.734^**^	0.294	0.954^**^	0.843^**^	0.494^*^	−0.355	0.662^**^	−0.707^**^
C20:4n-6	0.727^**^	−0.016	0.618^**^	−0.643^**^	−0.475^*^	−0.152	−0.552^*^	−0.722^**^	−0.291	0.245	−0.735^**^	0.560^**^

^*^P < 0.05; ^**^P < 0.01.

**Table 7:** **The relationship between estimated ELOVL6 and SCD1 activities, *de novo *synthesis index, C18:0, C20:2n-6, C20:4n-6 and C22:4n-7 in adipose tissue**

**Adipose tissue**	**Estimated ELOVL6 activity**				**Estimated SCD1 activity**				**C16:0/C18:2n-6**		**C18:0**	
	**C18:0/C16:0**		**C18:1/C16:1**		**C16:1**/**C16:0**		**C18:1**/**C18:0**					
	**Adult**	**Old**	**Adult**	**Old**	**Adult**	**Old**	**Adult**	**Old**	**Adult**	**Old**	**Adult**	**Old**
C20:2n-6	−0.497^**^	0.792^**^	−0.08	0.743^**^	0.338	−0.572^*^	0.613^**^	−0.551^*^	−0.492^*^	0.104	−0.657^**^	0.705^**^
C20:4n-6	−0.474^*^	0.485^*^	−0.264	−0.037	0.426^*^	−0.031	0.469^*^	−0.315	−0.675^**^	0.192	−0.653^**^	0.525^*^
C22:4n-7	−0.438^*^	0.561^*^	−0.041	0.279	0.161	−0.570^*^	0.495^*^	−0.730^**^	−0.13	0.546^*^	−0.476^*^	0.717^**^

^*^P < 0.05; ^**^P < 0.01.

### The expression of genes that influence fatty acid synthesis and catabolism

**Figure 7:**
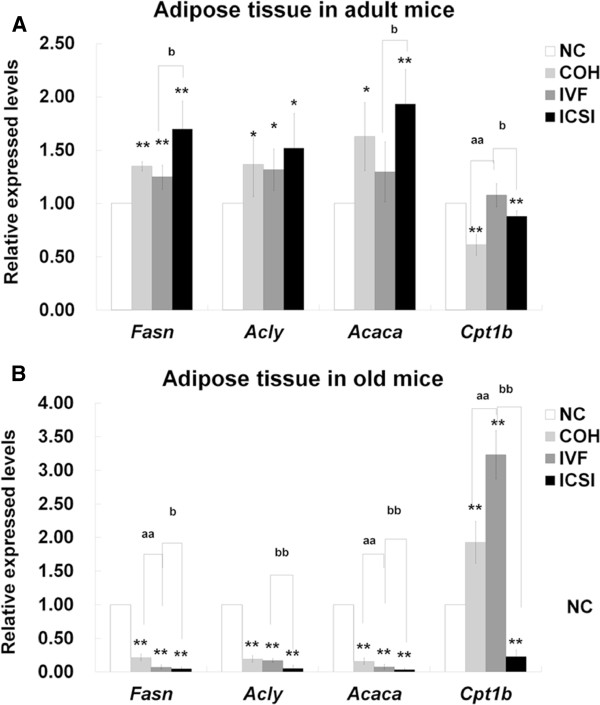
**The expression of genes related to fatty acid synthesis and catabolism in adipose tissue.** (**A**) The relative mRNA expression levels of *Fasn*, *Acly*, *Acaca* and *Cpt1b* in adult adipose tissue. (**B**) The relative mRNA expression levels of *Fasn*, *Acly*, *Acaca* and *Cpt1b* in old adipose tissue. ^*^P < 0.05 *vs*. NC group; ^**^P < 0.01 *vs*. NC group; ^aa^*P* < 0.01 *vs*. COH group; ^b^*P* < 0.05 *vs*. IVF group; ^bb^*P* < 0.01 *vs*. IVF group.

**Figure 8:**
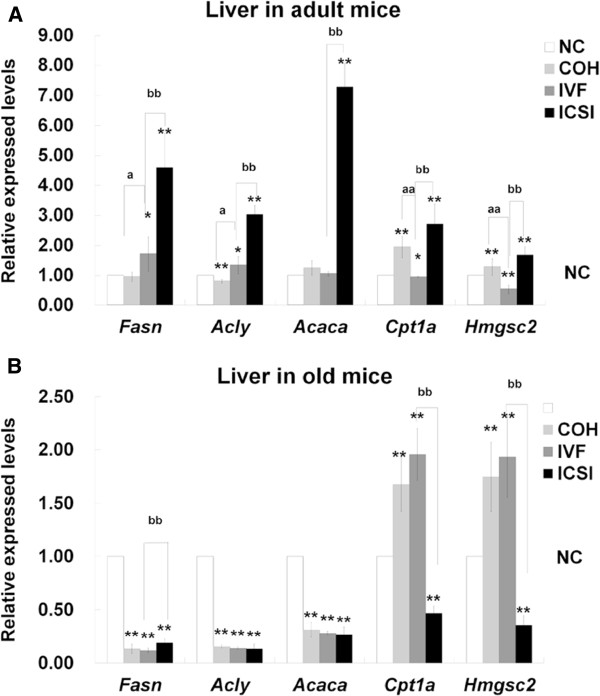
**The expression of genes related to fatty acid synthesis and catabolism in liver.** (**A**) The relative mRNA expression level of *Fasn*, *Acly*, *Acaca*, *Cpt1a* and *Hmgsc2* in adult liver. (**B**) The relative mRNA expression level of *Fasn*, *Acly*, *Acaca*, *Cpt1a* and *Hmgsc2* in old liver. ^*^P < 0.05 *vs*. NC group; ^**^P < 0.01 *vs*. NC group; ^a^*P* < 0.05 *vs*. COH group; ^aa^*P* < 0.01 *vs*. COH group; ^bb^*P* < 0.01 *vs*. IVF group.

In adult adipose tissue, the expression levels of *Fasn*, *Acly* and *Acaca* in three ART groups were higher than those in NC group, except *Acaca* in IVF group. *Cpt1b* in COH and ICSI group was significantly lower than that in NC group. Compared with COH group, *Cpt1b* was enhanced in IVF group. ICSI group contained higher levels of *Fasn* and *Acaca* and lower level of *Cpt1b* than IVF group (Figure 7A). In old adipose tissue, the expressions of *Fasn*, *Acly* and *Acaca* in ART groups significantly decreased when compared with NC group. *Cpt1b* was up-regulated in COH and IVF group and down-regulated in ICSI group in comparison with NC group. *Fasn* and *Acaca* in IVF group were expressed at lower level than those in COH group, while *Cpt1b* were expressed at higher level. The expressions of all the four genes in ICSI group were reduced in comparison with those in IVF group (Figure 7B). When compared with adult adipose tissue, increased *Fasn*, *Acly* and *Acaca* and decreased *Cpt1b* were found in old NC mice. The expression of *Fasn* and *Acaca* decreased in old COH and IVF group. All the four genes had lower expression levels in old ICSI group (Figure 8).

**Figure 9:**
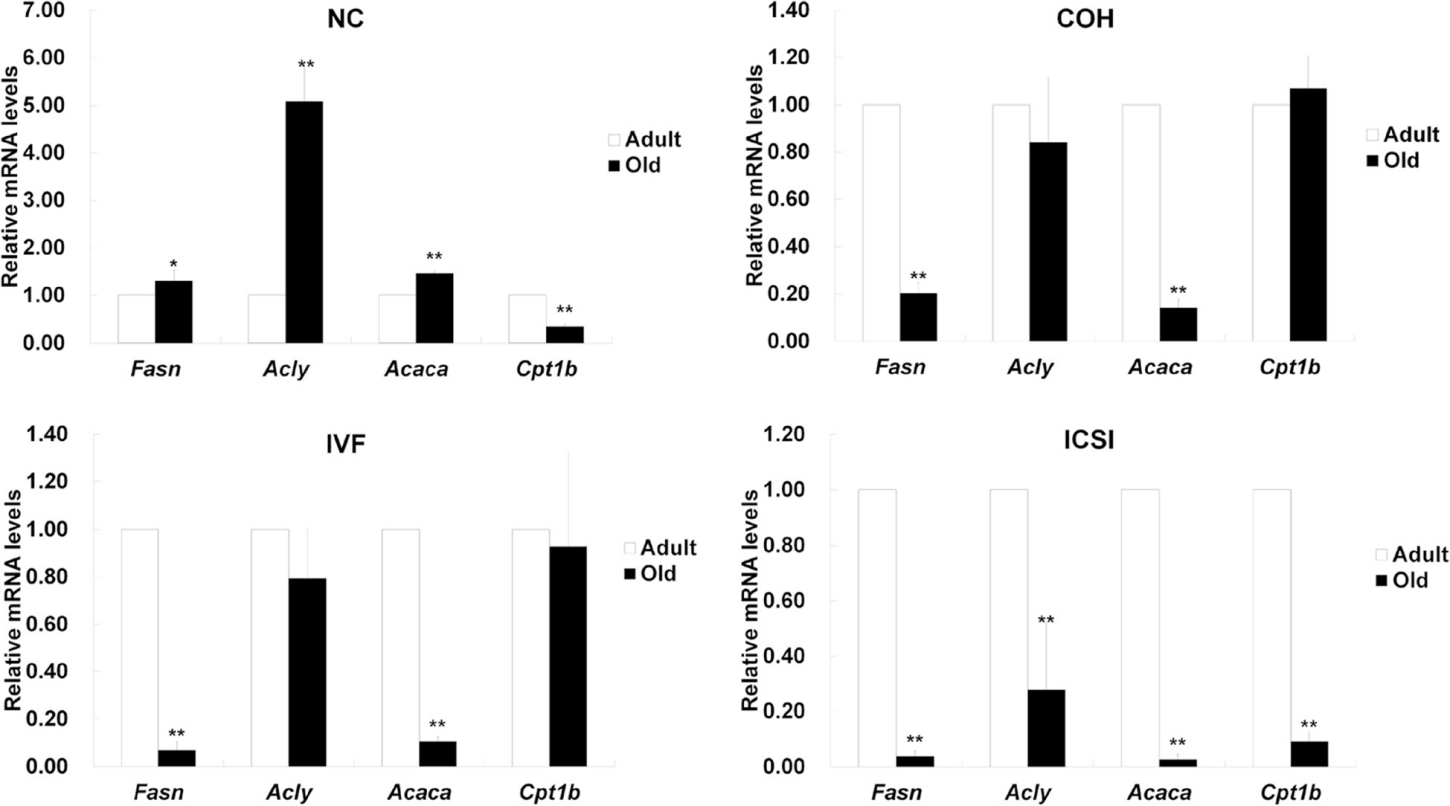
The comparison of the expression of *Fasn, Acly, Acaca *and*Cpt1b *in adipose tissue between adult and old mice.^*^P < 0.05 *vs*. adult group; ^**^P < 0.01 *vs*. adult group.

**Figure 10:**
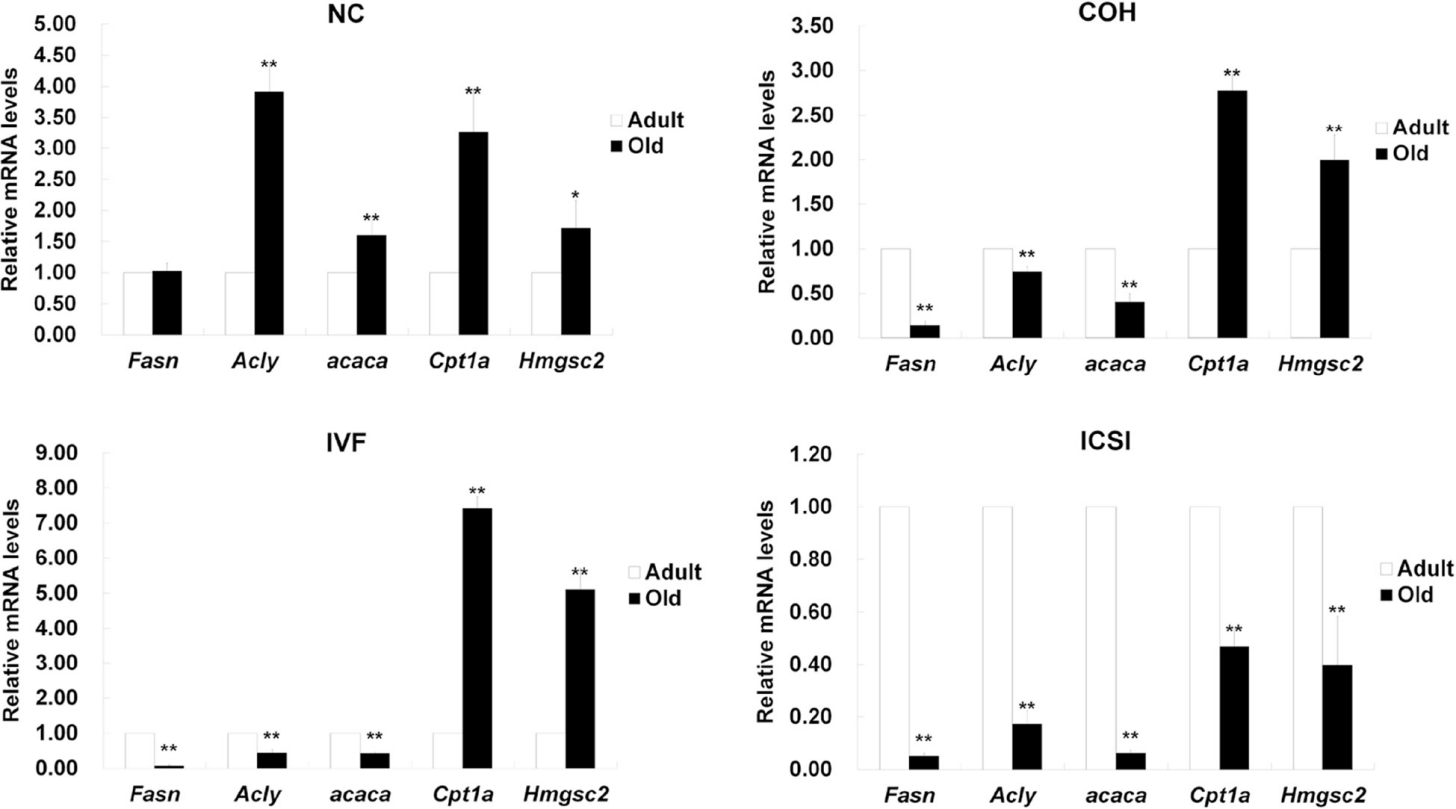
The comparison of the expression of *Fasn, Acly, Acaca, Cpt1a *and *Hmgsc2 *in liver between adult and old mice.^*^P < 0.05 *vs*. adult group; ^**^P < 0.01 *vs*. adult group.

In adult liver, compared with NC group, *Acly* was down-regulated and *Cpt1a* was up-regulated in COH group. *Fasn* and *Acly* had higher expressions, while *Cpt1a* and mitochondrial HMG-CoA synthase 2 (*Hmgsc2*) had lower expressions in IVF group. All the five genes in ICSI group showed increased expressions in comparison with NC group. Compared with COH group, the gene expression levels of *Fasn* and *Acly* were higher while *Cpt1b* and *Hmgsc2* were lower in IVF group. All these five genes in ICSI group had higher expressions than those in IVF group (Figure 9A). In old liver, compared with NC group, *Fasn*, *Acly* and *Acaca* were reduced in all three ART groups. *Cpt1a* and *Hmgsc2* were increased in COH and IVF group, but decreased in ICSI group. In ICSI group, *Fasn* was up-regulated and *Cpt1a* and *Hmgcs2* were down-regulated when compared with IVF group (Figure 9B). In the comparison between two age groups, the expressions of *Acly*, *Acaca*, *Cpt1a* and *Hmgcs2* were higher in old NC liver than those in adult liver. COH and IVF had lower expressions of *Fasn*, *Acly* and *Acaca* and higher expressions of *Cpt1a* and *Hmgsc2* in old liver. All the five genes were down-regulated in ICSI group with age (Figure 10).

## Discussion

Fatty acid composition in tissues can be changed with many factors [37]. The mice with the same age, sex and genetic background were chosen in this study, and the diet was also unified to secure difference data from different groups have a good comparability. The four predominant fatty acids (C16:0, C18:0, C18:1n-9 and C18:2n-6) and their content in the adipose tissue was quite similar to the fatty acid profile in the mouse diet, which support the main functions of adipose tissue in reserving energy and fatty acid storage. Fatty acid composition of dietary fat influences the lipid composition of adipose tissue [38,39]. Previous studies have shown that the molecular species composition of triacylglycerides in mammalian adipose tissues broadly reflected that of dietary triacylglycerides [40,41], which was further supported by our data. In contrast, the fatty acid composition in the liver and testis was obviously different. Specifically, both the liver and the testis contain significantly higher levels of C16:0 and C18:0, but lower levels of C18:1n-9 and C18:2n-6. The liver plays a key role in lipid metabolism, which is active in *de novo* fatty acid synthesis and ketogenesis [42,43]. The synthesis of SFAs in the testis supplies the individual requirements independent of dietary changes [44].

In the adult testis, compared with NC group, only three fatty acids were significantly different in individual ART group. The sum of SFAs, MUFAs and PUFAs showed no significant difference between the four groups, which indicated that the ART operations probably exert the minimal influence on the fatty acid composition in the testis. However, the fatty acid profiles in the adipose tissue and liver varied in adult mice between NC, COH, IVF and ICSI group. Compared with the NC mice, ART mice have significantly lower levels of PUFAs and higher levels of SFAs. The decrease of PUFAs mainly resulted from C18:2n-6. Antonini FM, Bucalossi A, Petruzzi E, Simoni R, Morini PL and D'Alessandro A [45] have reported that the fatty acid composition of adipose tissue was associated with atherosclerosis and diabetes. Compared to normal people, men with atherosclerosis had lower levels of C18:2n-6 in their adipose tissue. Unlike the adipose tissue, the alteration of fatty acid profile in the liver of ART mice was much more complex. C16:1n-7, one of the increased MUFAs in ICSI group, was reported as an important signaling lipid hormone newly designated as “lipokine”, which could stimulate the activity of muscle insulin action and influence fat deposition in the liver [46].

To detect the long-term effect of ART treatments on fatty acid metabolism, the fatty acid composition in old mice were also analyzed. In our results, the SFAs in adipose tissue increased with age and MUFAs decreased with age. In contrast, hepatic PUFAs were down-regulated in old mice. These changes were coincident with previous studies [47,48]. However, the changes of fatty acid composition between the four groups in old mice were different from those in adult mice. Compared with NC group, COH and IVF mice showed much more obvious difference than ICSI group. It is probably ART induced inconsistent changes of fatty acid composition during aging.

Since the content of SFAs, MUFAs and PUFAs can be regulated by many desaturases and elongases, and then the activities of these enzymes were analyzed between NC group and ART groups. The results showed the activities of fatty acid metabolism-related enzymes can also be influenced by both ART treatments and aging. According to the correlation analysis, SFAs (C16:0 in liver and C18:0 in adipose tissue) was closely associated with the relationship between fatty acid content and the lipid metabolism-related enzyme activities, which suggested that the variation of SFAs content may be the crucial step for regulating fatty acid composition.

Besides desaturases and elongases, lipogenic and lipolytic enzymes also participate in the regulation of fatty acid composition. FAS, ACLY and ACACA are key enzymes in *de novo* fatty acid synthesis, while CPT1A and CPT1B regulate the oxidation of fatty acids in liver and adipose tissue respectively. HMGCS2 determines the first reaction of ketogenesis. The lipid accumulation state can be reflected by the expression of fatty acid synthesis and catabolism-related genes. The increase in fatty acid synthesis, together with the decrease of fatty acid oxidation implied lipid accumulation [29,30]. We concluded the lipid accumulation state in each ART model and each treatment based on the expression of fatty acid synthesis and catabolism-related genes in Tables 8 and 9. Owing to the inconsistent expression of these genes in each mice model of two age groups, the lipid accumulation state was different. Likewise, we found the lipid accumulation state was generally identical to the changes of SFAs content in both adipose tissue and liver (that is, during the comparison between two groups, the increased lipogenesis and decreased lipolysis accompanied by the up-regulation of SFAs and vice versa). Therefore, the different gene expression of lipogenic and lipolytic enzymes can partly explain the change of fatty acid composition in adult and old ART mice and the variation of SFAs content might also be the crucial step. Interestingly, compared with NC adult adipose tissue, the enhanced lipid accumulation didn’t up-regulate the SFAs content in old mice. It was possible that the total lipid content in adipose tissue had changed with age.

**Table 8:** The conclusion of lipid accumulation state in adipose tissue

**Adipose tissue**		**Adult**			**Old**		
		**Lipogenesis**	**Lipolysis**	**lipid accumulation**	**Lipogenesis**	**Lipolysis**	**lipid accumulation**
COH	Superovulation	↑	↓	↑	↓	↑	↓
	In vitro culture	_	↑	↓	↓	_	↓
IVF		**↑**	**_**	**↑**	**↓**	**↑**	**↓**
	Mechanical stimulation	↑	↓	↑	↓	↓	?
ICSI		**↑**	**↓**	**↑**	**↓**	**↓**	**?**

According to the expression of fatty acid synthesis and lipolysis genes, we concluded that the accelerated lipid accumulation derived from increased lipogenesis genes or decreased lipolysis genes, while the opposite expression level implied reduced lipid accumulation.

**Table 9:** The conclusion of lipid accumulation state in liver

**Liver**		**Adult**			**Old**		
		**Lipogenesis**	**Lipolysis**	**lipid accumulation**	**Lipogenesis**	**Lipolysis**	**lipid accumulation**
COH	Superovulation	↓	↑	↓	↓	↑	↓
	In vitro culture	↑	↓	↑	↓	↑	↓
IVF		↑	↓	↑	↓	↑	↓
	Mechanical stimulation	↑	↑	?	↑	↓	↑
ICSI		**↑**	**↑**	**?**	**↓**	**↓**	**?**

According to the expression of fatty acid synthesis and lipolysis genes, we concluded that the accelerated lipid accumulation derived from increased lipogenesis genes or decreased lipolysis genes, while the opposite expression level implied reduced lipid accumulation.

It has been recognized that gonadal hormone can affect lipid metabolism [49], and *in vitro* culture may increase free oxygen radicals, which probably influences the lipid metabolism of embryos [50]. Mechanical stimulation has been reported to increase lipid-related second messengers [51]. Thus pairwise comparison between COH and IVF group and between IVF and ICSI group was also made to analyze the effect of *in vitro* culture and mechanical stimulation on fatty acid metabolism. According to our results, the effect of surperovulation, *in vitro* culture and mechanical stimulation on fatty acid metabolism changed with age in different tissues, among which the effect of superovulation and *in vitro* culture induced the reduced lipid accumulation in both adipose tissue and liver of old mice. Fatty acids function as energy provision and signals for metabolic regulation [52,53]. Although little research has been involved in the effect of decreased lipid accumulation, these changes must have potential effect on the health of old ART mice.

## Conclusion

Our studies for the first time concentrated on the lipid metabolism in ART male offspring. Fatty acid composition in the adult testis showed little difference between NC and ART groups, while obvious alteration was found in adipose tissue and liver. The differences of fatty acid composition between the four groups in old mice were different from those in adult mice. The effect of ART treatments on fatty acid metabolism changed with age in different tissues, and varied activities and expressions of fatty acid metabolism-related enzymes were the main regulation methods. The alteration of SFAs content may be the crucial step for regulating fatty acid profiles. Although the decreased lipogenesis and increased lipolysis were shown in adipose tissue and liver of COH and IVF old mice, the effects of these changes need further investigation. Our studies proved the influence of ART on fatty acid composition and the expression of lipid metabolism-related genes, which could provide a good reference for future investigation of ART safety.

## Methods

### ART models

All the protocols used in our investigation were approved by the Animal Care Ethics Committee of Zhejiang University. All C57BL/6J mice (6–8 weeks old) were randomly divided into four groups (NC/COH/IVF/ICSI groups) and raised under a standard environment (room temperature: 23 ± 1°C; humidity: 55 ± 5%) with a 12 hours light-dark cycle.

#### COH model

Female mice were injected with 7.5 IU pregnant mare serum gonadotrophin (PMSG) (GEN’s, Hangzhou, China) and 7.5 IU human chorionic gonadotrophin (hCG) (GEN’s, Hangzhou, China) 48 hours later. After superovulation, the female mice were naturally mated with male mice. COH embryos at the 2-cell stage were obtained from pregnant mice (appearance of a vaginal plug was designated as 0.5 days) at 1.5 days and transferred to the oviducts of pseudopregnant ICR mice. The COH mice were born after approximately 3 weeks.

#### IVF model

Female mice were firstly injected with 7.5IU PMSG and 7.5 IU hCG 48 hours later. MII oocytes were obtained 14 hours after hCG injection. IVF-fertilized oocytes were obtained after 6 hours of co-incubation of MII oocytes with sperm, and the fertilization procedure was performed in human tubal fluid (HTF) medium with 10% serum substitute supplement (SSS) (Irvine Scientific). The IVF-fertilized oocytes were cultured in 10% SSS G1 medium (Vitrolife). IVF embryos at the 2-cell stage were transferred to the oviducts of pseudopregnant ICR mice, and the IVF mice were born after approximately 3 weeks.

#### ICSI model

MII oocytes were obtained after the PMSG-hCG procedure. ICSI injection was operated using an Olympus X71 inverted microscope with PIEZO (PrimeTech, Ibaraki, Japan) and micromanipulators (Narishige, Tokyo, Japan) to obtain ICSI-fertilized oocytes. The fertilization procedure for ICSI was performed in warmed, HEPES-buffered, modified human tubal fluid medium (mHTF) (Irvine Scientific). ICSI-fertilized oocytes were cultured in 10% SSS G1 medium, and ICSI embryos at the 2-cell stage were transferred to the oviducts of pseudopregnant ICR mice, and the ICSI mice were born after approximately 3 weeks.

The liver, adipose tissue (abdominal adipose), and testis were obtained from three-month old (adult) and 18-month old (old) male mice (each group contained 5 individuals that were not siblings). All tissues were stored at -80°C for further lipid and RNA extraction. All the experiments below were replicated 3 times.

### Lipid extraction

Approximately 0.1 gram of sample (liver, abdominal adipose tissue, testis, and mice diet (Slac, Shanghai China, product number: Q/Scac01-2010)) was put into a tube containing heptadecanoic acid (0.2 mg/ml, Sigma) as an internal standard, and the lipid extraction from the tissues was performed using chloroform-methanol methods, which was modified based on the procedure used by Wang D-H, Chen Z-J, Jiang Y-Y, Zhou H and Yang W-X [54].

### Fatty acid transesterification

Transesterification was performed in 1 ml of 2% sulfuric acid-methanol (v/v) at 60°C for 1 hour, and then 2 ml hexane was added to promote fatty acid ester extraction. Subsequently, the fatty acid ester extraction was washed three times with 2 ml H_2_O each time. Moreover, anhydrous sodium sulfate was used to absorb the residual water. Ultimately, the hexane phase was obtained and dried using nitrogen gas, and the fatty acid methyl ester was dissolved in 0.5 ml hexane.

### GC-MS and fatty acid analysis

The fatty acid methyl esters were analyzed by gas chromatography-mass spectrometry (GC-MS) on a Thermo Focus GC/DSQII-MS with a hydrogen flame ionization detector and an HP-5-MS column (30 m × 0.25 mm × 0.25 μm). Helium served as the carrier gas, and 1μl sample was loaded when the injection temperature was 260°C. The column temperature program was set as follows: 140°C for 2 min; 140°C to 170°C at 4°C/min; 170°C for 1 min; 170°C to 240°C at 3.5°C/min; 240°C for 12.5 min; and 240°C to 260°C at 12°C/min. The detector temperature was 260°C. Fatty acid methyl esters were identified using MS databases.

### Quantitative real-time RT-PCR (qRT-PCR)

Total RNA from the liver and adipose tissue was extracted with Trizol (Invitrogen), and the cDNA was synthesized using purified total RNA and the SYBR PrimeScript™ RT-PCR Kit (Takara). Then, qRT-PCR was performed using the SYBR-Green I kit (Takara) in an ABI 7900 thermocycler [55]. *Gapdh* served as an internal control for analyzing gene expression. The primer sequences for qRT-PCR were shown in Additional file 2: Table S2.

### Analytical methods of fatty acids

*De novo* synthesis index were reflected by C16:0/C18:2 [56]. C18:0/C16:0 and C18:1/C16:1 were used to estimate the activities of EOLVL6, and both C18:1/C18:0 and C16:1/C16:0 were used to estimate the activities of SCD1 [16].

### Statistical analysis

SPSS 16.0 (SPSS Inc., Chicago, IL, USA) was used for the statistical evaluation. The qRT-PCR data were analyzed by the comparative CT method. One-way ANOVAs (Dunnett’s test) followed by the independent-sample t test was used for analyzing the alterations of fatty acid composition, the activities of fatty acid metabolism-related enzymes and gene expression. The correlation between the fatty acids proportion and the activities of fatty acid metabolism-related enzymes was analyzed by the Pearson correlation coefficient. The confidence interval was 95%.

## Abbreviations

ACACA: Acetyl-coenzyme A carboxylase alpha; ACLY: ATP citrate lyase; ART: Assisted reproductive technologies; COH: Controlled ovarian hyperstimulation; CPT: Carnitine palmitoyltransferase; DHA: Docosahexaenoic acid; ELOVL6: ELOVL Family Member 6; FAS: Fatty acid synthase; FSH: Follicle-stimulating hormone; GC-MS: Gas chromatography-mass spectrometry; HCG: Human chorionic gonadotrophin; HMGCS: Mitochondrial HMG-CoA synthase; HTF: Human tubal fluid; ICSI: Intracytoplasmic sperm injection; IVF: *In vitro* fertilization; MII: Metaphase II; MUFAs: Monounsaturated fatty acids; N_2_: Nitrogen gas; PMSG: Pregnant mare serum gonadotropin; qRT-PCR: Quantitative real time RT-PCR; SCD1: Stearoyl-CoA desaturase-1; SFAs: Saturated fatty acids; SSS: Serum substitute supplement; PUFAs: Polyunsaturated fatty acids.

## Competing interests

The authors declare that there is no conflict of interest that could be perceived as prejudicing the impartiality of the research reported.

## Authors' contributions

LYW participated in the design of the study, carried out the molecular biological studies and drafted the manuscript. FL, NW and LL carried out the establishment of ART mice models. YMZ, HYL, XRX, XMZ and HFH participated in the design of the study. YLC performed the statistical analysis. FJ conceived the study, participated in the design and helped to draft the manuscript. All authors read and approved the final manuscript.

## Supplementary Material

Additional file 1 **Table S1.** Relationship between SFAs, MUFAs, PUFAs, estimated ELOVL6 and SCD1 activities and *de novo* synthesis index. Click here for file

Additional file 2 **Table S2.** List of the primers used for qRT-PCR. Click here for file
