# Supplementary material for: Alteration of fatty acid metabolism in the liver, adipose tissue, and testis of male mice conceived through assisted reproductive technologies: fatty acid metabolism in ART mice
Source: Lipids Health Dis. 2013 Jan 23;12:5. doi: 10.1186/1476-511X-12-5 (PMC3570477; doi:10.1186/1476-511X-12-5)
Supplement: Additional file 1 — Table S1. Relationship between SFAs, MUFAs, PUFAs, estimated ELOVL6 and SCD1 activities and de novo synthesis index. [file 1476-511X-12-5-S1.doc]

**Table S1 Relationship between SFAs, MUFAs, PUFAs, estimated ELOVL6 and SCD1 activities and *de novo*** synthesis index

|  |  | **SFAs** | |  | **MUFAs** | |  | **PUFAs** | |
| --- | --- | --- | --- | --- | --- | --- | --- | --- | --- |
|  |  | **Adult** | **Old** |  | **Adult** | **Old** |  | **Adult** | **Old** |
| **Adipose tissue** | |  |  |  |  |  |  |  |  |
|  | C16:0/C18:2 | 0.947^**^ | 0.895^**^ |  | -0.101 | -0.276 |  | -0.874^**^ | -0.637^**^ |
| Estimated ELOVL6 activity | | |  |  |  |  |  |  |  |
|  | C18:0/C16:0 | 0.29 | 0.319 |  | -0.243 | -0.08 |  | -0.106 | -0.25 |
|  | C18:1/C16:1 | -0.091 | 0.143 |  | -0.012 | 0.234 |  | 0.099 | -0.451 |
| Estimated SCD1 activity | | |  |  |  |  |  |  |  |
|  | C16:1/C16:0 | -0.489^*^ | -0.627^**^ |  | 0.490^*^ | 0.235 |  | 0.118 | 0.394 |
|  | C18:1/C18:0 | -0.676^**^ | -0.760^**^ |  | 0.642^**^ | 0.486^*^ |  | 0.188 | 0.227 |
| **Liver** | | |  |  |  |  |  |  |  |
|  | C16:0/C18:2 | 0.578^**^ | 0.886^**^ |  | 0.502^*^ | -0.403^*^ |  | -0.654^**^ | -0.492^**^ |
| Estimated ELOVL6 activity | | |  |  |  |  |  |  |  |
|  | C18:0/C16:0 | -0.326 | 0.562^**^ |  | -0.759^**^ | -0.158 |  | 0.727^**^ | -0.440^*^ |
|  | C18:1/C16:1 | 0.01 | 0.146 |  | -0.559^*^ | 0.522^**^ |  | 0.417 | -0.855^**^ |
| Estimated SCD1 activity | | |  |  |  |  |  |  |  |
|  | C16:1/C16:0 | 0.05 | -0.830^**^ |  | 0.777^**^ | 0.373 |  | -0.609^**^ | 0.467^*^ |
|  | C18:1/C18:0 | 0.146 | -0.864^**^ |  | 0.969^**^ | 0.876^**^ |  | -0.799^**^ | -0.155 |

C16:0/C18:2 reflected *de novo* synthesis index. Estimated ELOVL6 activity was reflected by C18:0/C16:0 and C18:1/C16:1. Estimated SCD1 activity was reflected by C16:1/C16:0 and C18:1/C18:0. ^*^ *P*< 0.05; ^**^ *P*< 0.01.
