# Supplementary material for: Alteration of fatty acid metabolism in the liver, adipose tissue, and testis of male mice conceived through assisted reproductive technologies: fatty acid metabolism in ART mice
Source: Lipids Health Dis. 2013 Jan 23;12:5. doi: 10.1186/1476-511X-12-5 (PMC3570477; doi:10.1186/1476-511X-12-5)
Supplement: Additional file 2 — Table S2. List of the primers used for qRT-PCR. [file 1476-511X-12-5-S2.doc]

**Table S2** List of the primers used for qRT-PCR

| **Gene name** | **GenBank Accession** | **Primer sequence(5'to3')** | **Amplicon Size (bp)** |
| --- | --- | --- | --- |
| *Acaca* | NM_133360 | 5'-CCGATTCATAATTGGGTCTGTGT-3' | 156 |
|  |  | 5'-CCATCCTGTAAGCCAGAGATCC-3' |  |
| *Acly* | NM_134037 | 5'-CAGCCAAGGCAATTTCAGAGC-3' | 195 |
|  |  | 5'-CTCGACGTTTGATTAACTGGTCT-3' |  |
| *Cpt1a* | NM_013495 | 5'-GGGTCGAAAGCCCATGTTGTA-3' | 142 |
|  |  | 5'-CAGTGCTGTCATGCGTTGGA-3' |  |
| *Cpt1b* | NM_009948 | 5'-GCACACCAGGCAGTAGCTTT-3' | 107 |
|  |  | 5'-CAGGAGTTGATTCCAGACAGGTA-3' |  |
| *Fasn* | NM_007988 | 5'-GGAGGTGGTGATAGCCGGTAT-3' | 140 |
|  |  | 5'-TGGGTAATCCATAGAGCCCAG-3' |  |
| *Hmgcs2* | NM_008256 | 5'-TCGAGGGCATAGATACCACCAAC-3' | 127 |
|  |  | 5'-CCGCGCTTCAGTTCAGTGTC-3' |  |
